# Learning by task repetition enhances object individuation and memorization in the elderly

**DOI:** 10.1038/s41598-020-75297-x

**Published:** 2020-11-17

**Authors:** Chiara F. Tagliabue, Sara Assecondi, Giulia Cristoforetti, Veronica Mazza

**Affiliations:** 1 Center for Mind/Brain Sciences (CIMeC), University of Trento, Corso Bettini 31, 38068 Rovereto, TN Italy; 2 School of Psychology and Centre for Human Brain Health (CHBH), University of Birmingham, Birmingham, UK; 3 Department of Experimental Psychology, University of Ghent, Ghent, Belgium

**Keywords:** Cognitive ageing, Cognitive neuroscience, Learning and memory

## Abstract

A decline in visuospatial Working Memory (vWM) is a hallmark of cognitive aging across various tasks, and facing this decline has become the target of several studies. In the current study we tested whether older adults can benefit from task repetition in order to improve their performance in a vWM task. While learning by task repetition has been shown to improve vWM performance in young adulthood, little is known on whether a similar enhancement can be achieved also by the aging population. By combining different behavioral and electrophysiological measures, we investigated whether practicing a specific task (delayed match-to-sample judgement) over four consecutive sessions could improve vWM in healthy aging, and which are the neurophysiological and cognitive mechanisms modulated by learning. Behavioral data revealed that task repetition boosted performance in older participants, both in terms of sensitivity to change (as revealed by d’ measures) as well as capacity estimate (as measured by k values). At the electrophysiological level, results indicated that only after task repetition both target individuation (as evidenced by the N2pc) and vWM maintenance (as reflected by the CDA) were modulated by target numerosity. Our results suggest that repetition learning is effective in enhancing vWM in aging and acts through modifications at different stages of stimulus processing.

## Introduction

Cognitive aging is associated with a decline in the domain of visuospatial working memory (vWM)^1–3^, namely the ability to simultaneously maintain and act on visual stimulus material^4^. Changes to various mechanisms operating at different stages of stimulus processing have been suggested to explain the age-related reduction in vWM. Three main changes have been proposed: (1) Primary changes in early perception (in terms of lower perceptual ability^5–8^ or in terms of reduced processing speed at early perceptual stages^9,10^, which do not allow the elderly to generate accurate representations of memoranda; (2) A decreased efficiency of attention selection^9,11–16^, so that older individuals cannot discard irrelevant information that is later encoded in the WM buffer; (3) A loss in the capacity of the short-term storage^17–19^ where to-be-remembered material is retained.

Since vWM is involved in the execution of several tasks that affect everyday activities^20^, facing its age-related decline has become the target of several studies. In the current study we tested whether older adults can benefit from task repetition in order to improve their performance in a vWM task. We relied on electrophysiological (EEG) measures to maximize the possibility to isolate which mechanisms (if any) underlying task execution are influenced by learning via task repetition.

Previous studies on young adults have indicated that repetition of different vWM tasks (e.g. delayed match-to-sample, *n*-back) produces positive outcomes, both after a variable number of sessions performed in different days^21^ (see also^22,23^ for extensive practice during more complex training intervention or electrical stimulation studies), and within the same session^24–27^. These enhancements are usually visible in terms of increase in accuracy and/or speed, and have been associated with a modulation of the neural activity at different stages of stimulus processing (e.g. encoding, categorization, depending on the task chosen^21,24–27^).

The literature on learning by task repetition in older participants is scarce. In most of the studies on older individuals, repetition practice of a specific vWM task is exploited over an extended period of time for a different experimental aim, namely to provide evidence in favor of transfer gain to untrained tasks^28–30^. However, since the decline in vWM is a distinctive cognitive feature of aging^1–3^, investigating the short-term effects of task repetition in the elderly has two main advantages. First, it is a (relatively) simple and rapid way to evaluate whether a specific ability can be enhanced in older adults (and it may become a useful marker of an individual’s degree of residual cognitive flexibility^31^). Second, when coupled with the isolation of the specific mechanisms associated with vWM improvement, it may provide useful prompts for the development of longer-term cognitive interventions specifically targeting these mechanisms (see^32,33^ for a review on long-term WM trainings).

To investigate the effect of learning by task repetition, we capitalized on a delayed match-to-sample paradigm (DMTS). In the past decade, research on the electrophysiological correlates of vWM functioning has extensively used this task (e.g.^34–39^), which was in turn successfully adopted from neurophysiological studies on non-human primates^40–45^. Strong convergence has also been found between areas activated while performing the DMTS and the brain regions usually associated with the WM network (dorsolateral prefrontal cortex, superior frontal gyrus and posterior parietal cortex; for a recent meta-analysis see^46^). DMTS is particularly suitable for the aim of the present study for two reasons. First, differently from other tasks probing vWM functioning (e.g. *n*-back), the defined sequence of events in the DMTS task allows for the temporal isolation of (at least some) specific mechanisms subserving vWM. Indeed, in this task participants first have to encode the stimuli and then deploy an attention-based selective mechanism that allows them to individuate the target elements. Once the relevant items are selected, they are retained in a vWM buffer, where they can ultimately be compared to a probe array^14,15,47,48^. In terms of EEG measurements, some event-related responses have been widely identified as markers of the cognitive mechanisms implicated in DMTS: (1) the N1, an early visual component of perceptual encoding^49,50^; (2) the N2 posterior-contralateral (N2pc), an attention-related response of target individuation^51–53^; (3) the contralateral delay activity (CDA), a late sustained component indexing active item maintenance in the vWM buffer^39^. Crucially, the amplitude of both N2pc and CDA is sensitive to the number of items that need to be simultaneously individuated/memorized and reaches a plateau that is in line with the behavioral performance of the individual^39^.

Second, together with concurrent EEG recordings, DMTS judgements have recently been used in the aging population^13–15,54–56^, to identify which cognitive mechanisms are selectively affected by, or resistant to, senescence. The most consistent result in these studies is an age-related reduction both in the amplitude and in the modulation of the CDA as a function of memory load^13–15,55,56^, which is thought to reflect the lower vWM capacity of older compared to young adults. Moreover, in DMTS tasks where targets were presented among distractors^13–15^ (see also^56^ for targets and distractors in opposite hemifields), an impaired or delayed distractor suppression mechanism was associated in the elderly with changes in the CDA^13,14^, indicating that older participants were retaining also irrelevant items. Changes were also found in the N2pc^15^, and were linked to an age-related deficit at an earlier cognitive stage, namely during target selection.

In the present study, young and older participants performed a lateralized DMTS task over four consecutive days, with electrophysiological recordings in the first and last session. We investigated three behavioral indexes and three electrophysiological measures typically elicited by DMTS tasks. Specifically, to increase our knowledge of the effect of task repetition on behavioral performance we considered three different measures: (1) the sensitivity threshold (d’^57^), i.e. the ability to discriminate signal from noise; (2) the decision bias (c^57^), i.e. the response approach adopted by the participant; (3) the estimate of the vWM capacity limit (k^58,59^). This allowed us to test whether any age-related effect of learning (if present) would be reflected in a shift of response bias or in a genuine change of discrimination sensitivity and/or vWM capacity limit.

At the electrophysiological level, we considered the following measures associated with the mechanisms usually engaged in multiple target processing, namely: the N1 (as a proxy of early perceptual processing), the N2pc (target individuation) and the CDA (WM maintenance). We were interested in how task repetition influenced the load-related modulations of these EEG responses, as such modulation is indicative of the efficiency in discriminating the to-be-memorized items.

The overarching aim of the study was to obtain a deeper understanding of the changes following learning by task repetition, with a special focus on aging individuals. At the behavioral level, we asked whether repetition practice would selectively improve vWM capacity or it would lead to a general enhancement encompassing also the discrimination ability. At the electrophysiological level, we considered the presence of a significant difference between memory loads (i.e. load-related modulation) as a proxy of the efficiency in discriminating the memoranda. Therefore, we considered that if such modulation is present only after task repetition, it is indicative of a learning effect (i.e. improved neural efficiency in discriminating the targets). By making use of the detailed temporal information present in EEG recordings, we additionally tested for the presence of such modulation across the different levels of stimulus processing, namely early perception (N1), attentive selection (N2pc) and memory retention (CDA). If learning exclusively influences the capacity to maintain elements in memory, we expect to observe different patterns of load-related modulations in the two sessions only in the CDA latency. In contrast, if repetition-learning also influences earlier perceptual or attentional mechanisms, a different pattern of load-related modulations should be evident in the N1 and/or in the N2pc, with a potential cascade effect on the CDA.

## Materials and methods

### Participants

Sample size was determined a priori, based on previous practice/training studies^29,60–62^. Twenty-eight healthy older adults and 25 healthy young adults participated in the study. Data from one young and four older adults were excluded from the analyses due to excessive noise during EEG recording. The final sample consisted of 24 healthy older adults (13 women; age range: 63–79; mean age ± standard deviation = 70.21 ± 4.97; mean education ± standard deviation = 12.21 years ± 2.10) and 24 healthy young adults (12 women; age range: 19–32; mean age ± standard deviation = 24.08 ± 3.01; mean education ± standard deviation = 15.79 years ± 1.61). All reported normal or corrected-to-normal vision and a negative history of neurological or psychiatric disorders. Written informed consent to participate in the study was obtained prior to testing. The study was approved by the Ethics Committee of the University of Trento and conducted in accordance with the 2013 Declaration of Helsinki.

### Neuropsychological testing

Older adults were administered a battery of neuropsychological tests in order to assess their cognitive status. The duration of the neuropsychological assessment was approximately 50 minutes. In order to avoid fatigue, the assessment was split and administered prior to each of the two intermediate sessions (i.e. Session 2 and Session 3; see Experimental Sessions). The exclusion criterion was set to more than one test score below the cut-off values. No data were excluded for this reason. The mean scores for each cognitive test are reported in Table 1.

**Table 1:** Mean raw and correct scores (standard deviation in parentheses) at each neuropsychological test.

Neuropsychological test	Mean raw score (SD)	Mean correct score (SD)	Cutoff
MMSE^98^	28.54 (1.44)	28.24 (1.58)	≤ 23.80
RAVLT immediate recall^99^	50.46 (10.89)	53.69 (10.56)	≤ 28.52
RAVLT delayed recall^99^	10.88 (3.43)	11.99 (3.36)	≤ 4.68
Digit span forward^100^	5.5 (0.91)	5.65 (0.86)	< 4.26
Digit span backward^100^	4.33 (1.03)	4.43 (1.05)	< 2.65
RCPM 47^101^	33 (3.93)	34.28 (3.21)	≤ 18
Attentive matrices^102^	55.04 (3.93)	54.09 (4.34)	≤ 30
TMT B-A^103^	47.71 (20.27)	17.46 (21.09)	> 186
ROCF copy^104^	33.25 (1.79)	34.24 (1.85)	≤ 28.87
ROCF recall^104^	16.79 (4.93)	19.69 (5.9)	≤ 9.46
Stroop reaction times^105^	18.57 (7.58)	10.84 (7.38)	≥ 36.92
Stroop errors^105^	1.2 (2.03)	0.55 (1.93)	≥ 4.24
Phonemic fluency^106^	40.96 (11.83)	39.32 (12.01)	< 17.35
Geriatric depression scale^107^	4.63 (3.35)	/	> 14

Cutoff scores indicate the value above/below which the cognitive performance is considered pathological.

*MMSE* mini mental state examination, *RAVLT* Rey’s auditory verbal learning test, *RCPM* Raven’s coloured progressive matrices, *TMT* trail making test, *ROCF* Rey–Osterrieth complex figure.

### Stimuli and procedure

Participants performed a lateralized delayed match-to-sample task^39^, identical to the one of a previous study^56^. Stimuli were colored and light grey dots (30 cd/m^2^, with a diameter of 1°; light grey dots RGB [150, 150, 150]), presented on a dark grey background (20 cd/m^2^; RGB [100, 100, 100]). For dots presented in the ‘relevant’ hemifield (targets) the colors selected were red (RGB [250, 0, 0]), blue (RGB [0, 20, 165]), yellow (RGB [250, 250, 0]), light green (RGB [0, 250, 0]) and purple (RGB [139, 58, 98]); colors used for the ‘irrelevant hemifield’ (distractors) were orange (RGB [255, 127, 0]), light blue (RGB [64, 224, 208]), dark green (RGB [34, 139, 34]), pink (RGB [255, 105, 180]) and brown (RGB [139, 69, 19]). Each stimulus array consisted of one, two or four colored dots independently presented in each hemifield together with light grey dots, resulting in the same or different number of colored dots across the two hemifields (all experimental conditions were counterbalanced across the two hemifields). Throughout the experiment, the total number of stimuli presented on the screen was kept constant (18 items in total: nine items for each hemifield, comprising colored + light grey dots). Stimulus arrays were arranged within an invisible 8 (rows) × 10 (columns) (13.8° × 16.4°) rectangular grid centered at the center of the screen, where a white fixation cross was present for the entire trial procedure. Stimulus positions were randomized on each trial, with the constraint that colored dots never appeared in the extreme rows and columns or in the columns closest to the fixation cross.

**Figure 1:**
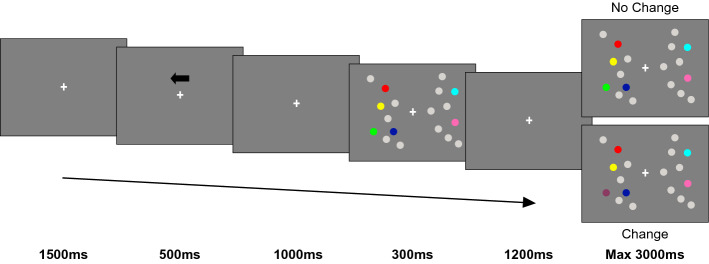
Trial sequence. An example of stimulus sequence with four targets in the left hemifield.

Participants sat in front of a 19-inch LCD monitor (resolution 1280 × 1024, refresh rate of 75 Hz, viewing distance of 85 cm) and performed a change detection task on lateralized stimuli (Fig. 1). At the beginning of each trial a fixation cross was presented for 1500 ms followed by a black arrow (3.3°) appearing above the central fixation cross for 500 ms. The arrow pointed randomly and with equal probability leftward or rightward, indicating the to-be attended hemifield (‘relevant hemifield’). The arrow cue was always valid. After an interval of 1 s, the memory array was presented for 300 ms, followed by a 1200 ms retention interval. Participants were instructed to memorize the colors of the stimuli in the cued ‘relevant hemifield (targets) and to ignore the stimuli in the ‘irrelevant hemifield’ (distractors). On 50% of the trials, the test array was identical to the memory array (i.e. no-change condition), while in the remaining 50% of the cases one target in the relevant hemifield changed color (i.e. change condition). Participants were informed that the colors of the distracters in the irrelevant hemifield never changed. Moreover, the locations of the colored dots never changed and, in the case of a change trial, the dot changing in color was a previously colored dot. The test array remained on the screen until response, or for a maximum of 3 s. Participants reported whether the probe differed or not with respect to the memory array. They used both hands, pressing with either their right or left index finger a designated key (letter M or C). Response assignment to each key (‘same’, ‘different’) was counterbalanced between subjects.

### Experimental sessions

The same task was performed over four consecutive days. The choice of the number of sessions was driven by the need to strengthen practice-related effects beyond test–retest gains. During the first and last day (hereafter called Session 1 and Session 4, respectively), participants completed a total of 720 trials (240 trials for each memory load—one, two, four) divided into 15 blocks of 48 trials each, after performing a practice block of 10 trials. In Session 1 and 4, the EEG signal was simultaneously recorded. During Session 2 and 3, the task was performed with a semi-adaptive procedure. Specifically, participants completed a total of 384 trials divided into eight blocks of 48 trials each. The total number of trials for each target numerosity depended on the accuracy obtained at each memory load during the previous day (Session 1 and Session 2 to determine the number of trials for Session 2 and Session 3, respectively)^23,63^, following the logic that conditions where participants were performing poorly (i.e. < 75% of accuracy) were augmented in trial number and vice-versa.

### EEG recordings and analysis

EEG was continuously recorded using 29 active electrodes with a sampling rate of 1000 Hz, a time constant of 10 s as low cut-off and 250 Hz of high-cutoff. AFz served as ground and the right mastoid as the on-line reference. Horizontal ocular movements were recorded using two electrodes placed on the outer canthi of both eyes. Electrode impedances were kept below 20 kΩ.

The continuous EEG signal was then processed off-line using EEGLAB^64^, ERPLAB^65^ and FieldTrip^66^. All the pre-processing steps were separately performed on each EEG session. Data were down-sampled to 250 Hz and filtered with a low-frequency cutoff of 0.1 Hz and a high-frequency cutoff of 40 Hz. Additionally, to remove the 50 Hz line noise, a notch band-pass filter (width: 2 Hz) was also applied. All channels were re-referenced to the average of the left and right mastoids. Independent component analysis (ICA; Infomax ICA algorithm^67^) was applied to the whole dataset for each session of each participant to manually identify and correct for eye blinks, saccades, muscle and cardiac activity. Epochs with correct responses were then segmented from − 200 to 900 ms relative to the onset of the memory array. Baseline correction was applied by subtracting the mean activity during a 200 ms pre-stimulus recording period. Beyond removing ICAs related to saccades, trials with originally uncorrected horizontal eye movement activity exceeding ± 50 µV (on HEOG channel, computed by subtracting the activity of the right from the left ocular channel) were removed^68^. Also, trials with any channel activity exceeding ± 80 µV were rejected. Finally, epochs were collapsed across change condition (change, no-change) and target side (left, right), to obtain activity contralateral and ipsilateral to the target, regardless of the actual cue direction/target position (i.e. for targets presented in the left hemifield, right channels were used to compute contralateral activity and left channels for ipsilateral activity, and vice-versa for targets in the right hemifield). For each session, three main conditions were extracted depending on the load (Load1, Load2, Load4). After pre-processing, for the Young group the mean number of epochs yielding correct responses retained for the average in Session 1 was 199.46 (83% of the total trials) for Load1, 196.33 (82%) for Load2 and 172.63 (72%) for Load4, while in Session 4 the mean number of epochs retained was 201.13 (84%) for Load1, 199.29 (83%) for Load2 and 173.88 (72%) for Load4. In the Old group, the mean number of epochs in Session 1 was 184.75 (77%) for Load1, 176.38 (73%) for Load2 and 135.83 (57%) for Load4, while in Session 4 there were 188.21 (78%) epochs for Load1, 186.38 (78%) for Load2 and 147.63 (62%) for Load4.

### Statistical analysis

Not all behavioral and electrophysiological variables satisfied the assumptions of normality and homogeneity of variances (see Supplementary Tables 1 and 2 for statistical details). Nonetheless, we decided to perform parametric tests, given that they prove to be robust to such violations (normality^69–72^; homogeneity of variances^73–75^).

#### Behavioral data

vWM capacity is usually computed using the highest memory load condition (e.g.^55^) which better represents the vWM limit of the participants. Therefore, for the analyses of all behavioral data we computed the indexes on the basis of Load4. We measured sensitivity (d’), response criterion (c^57^) and memory capacity (k, computed according to Pashler’s formula: k = [(hit rate – false alarm rate)/(1 – false alarm rate)] × load. Load refers to the number of colored target dots that participants had to remember, i.e. four^59,76^). Following previous studies^14,38,39,47,56^, hit rates were defined as ‘different’ responses in change conditions, while false alarms were ‘different’ responses in no-change trials. It follows that, in the present study, a conservative response criterion is defined as the tendency to answer ‘Same’ on change trials and is represented by positive values; vice-versa, a more liberal behavior was considered the tendency to answer ‘Different’ on no-change trials and is represented by negative values.

Due to the different number of trials delivered in Sessions 1 and 4 with respect to Session 2 and 3, separate statistical analyses were performed on Session 1 and Session 4 versus Session 2 and Session 3. Since Session 2 and 3 did not include the EEG recording, the main results will only be summarized. To investigate overall improvements between the first and last session, an analysis of variance (ANOVA) was then conducted on d’, c and k measures with Age (two levels: Young, Old) as between-subjects and Session (two levels: Session 1, Session 4) as within-subjects factor.

#### ERPs

Following previous studies^13,14,56^, to obtain a precise temporal evolution of the ERP components, the analysis was performed in three consecutive steps by identifying the temporal windows of interest without any a priori selection. This data-driven approach allowed us to take into account slight latency differences in the onset and development of the components that might be present between the two age groups.

First, for each participant of each group a single difference wave was computed by subtracting ipsilateral from contralateral-to-target activity^15^ collapsed across Load condition (i.e. one, two, four) and Session (i.e. Session 1 and 4) in a region of interest (ROI) comprising electrodes O1/2, P7/8 and PO7/8 (see^77^). Then, separately for young and older adults, we followed a moving window approach with a non-overlapping window length of 20 ms, from the onset of the memory array to 900 ms post-onset. For each time window, we used a one sample t-test to assess whether the mean amplitude in that time window differed from zero. A significant difference lasting for at least two consecutive time windows (i.e. 40 ms) was considered reliable. This procedure resulted in three distinct significant time ranges for each group (Young: 100–140 ms, 240–400 ms, 460–900 ms; Old: 140–180 ms, 220–300 ms, 440–900 ms). We then averaged the starting and ending time point respectively of the previously identified windows across groups, in order to obtain common time windows for both young and older participants for subsequent analysis. The final resulting latencies were 120–160 ms, 230–350 ms and 450–900 ms, roughly reflecting the ERP components usually found in the literature around those latencies: N1, N2pc and CDA.

Second, within each one of these latencies, the mean amplitude was computed for each load, session and age group and a main ANOVA was conducted with Age as between-subjects factor and Session, Load and Latency (three levels: N1, N2pc, CDA) as within-subjects factors.

Third, significant main or interaction effects resulting from the main ANOVA were then separately investigated in each session, latency and age group (via paired-samples t-tests, and comparing one vs two and two vs four target-trials for Load-related effects), again employing the 20-ms consecutive time window approach (and two consecutive significant time windows to consider a difference as reliable^56^).

For the ANOVAs on both behavioral and electrophysiological data, in case of violation of sphericity, Greenhouse–Geisser (when G–G epsilon < 0.75) or Huynh–Feldt (when G–G epsilon > 0.75) correction was used, and only adjusted p values are reported. All follow-up pairwise comparisons were conducted through t-tests and correction for multiple comparisons (adjusted p values are reported), in order to reduce Type 1 error, was performed using the False Discovery Rate (FDR) procedure^78^, also for the t-tests that were employed in the 20-ms moving window analyses. Effect sizes were computed as partial eta squared (η_p_^2^) for ANOVAs and Cohen’s d for t-tests^79^. For dependent-samples t-tests, the following Eq. (1) was used:

1 $$d=\frac{{\upmu }_{\Delta }}{{\sigma }_{\Delta }}$$

where μ_Δ_ is the mean difference between conditions and σ_Δ_ is the standard deviation of the difference. For independent-samples t-tests, the following Eq. (2) was used:

2 $$\mathrm{d}=\frac{{\upmu }_{1}-{\upmu }_{2}}{\sqrt{\frac{{\upsigma }_{1}^{2}+{\upsigma }_{2}^{2}}{2}}}$$

where μ_1_ is the mean of Group 1, μ_2_ the mean of Group 2, σ_1_ the standard deviation of Group 1 and σ_2_ the standard deviation of Group 2.

As behavioral and EEG data provide different information, we adopted a between-sessions approach for the behavioral measures, and a within-session approach for the EEG measures. As the absolute values of the behavioral indexes (i.e. d’, c, and k) have an intrinsic meaning (e.g. k = 3 indicates that the WM limit is around three elements), comparing them between sessions can actually quantify the amount of learning. In contrast, the amplitude values of the ERP components do not have a similar intrinsic meaning, therefore comparing them between sessions might lead to ambiguous results (i.e. a learning-related increase/decrease in amplitude has not a precise meaning, see for example^80^). We thus used a less ambiguous approach to investigate learning-related changes in the efficiency of the mechanisms subserving vWM, and tested for the presence, in each session, of a load-related modulation. Such modulation acts as a proxy of the efficiency in discriminating one vs two vs four items during the different processing stages (i.e. early perceptual processing—N1, selection—N2pc, retention—CDA).

## Results

In line with a previous study^56^, we found that numerical similarity (see Stimuli and Procedure section) between colored targets and colored distractors did not influence the performance (see Supplementary Information for further details). We therefore collapsed trials across number of distractors for further analysis.

### Sensitivity (d’)

#### Session 1 vs Session 4

The mixed ANOVA showed significant main effects of Session (*F*(1, 46) = 25.724, *p* < 0.001, η_p_^2^ = 0.359) and Age (*F*(1, 46) = 38.79, *p* < 0.001, η_p_^2^ = 0.457).

**Figure 2:**
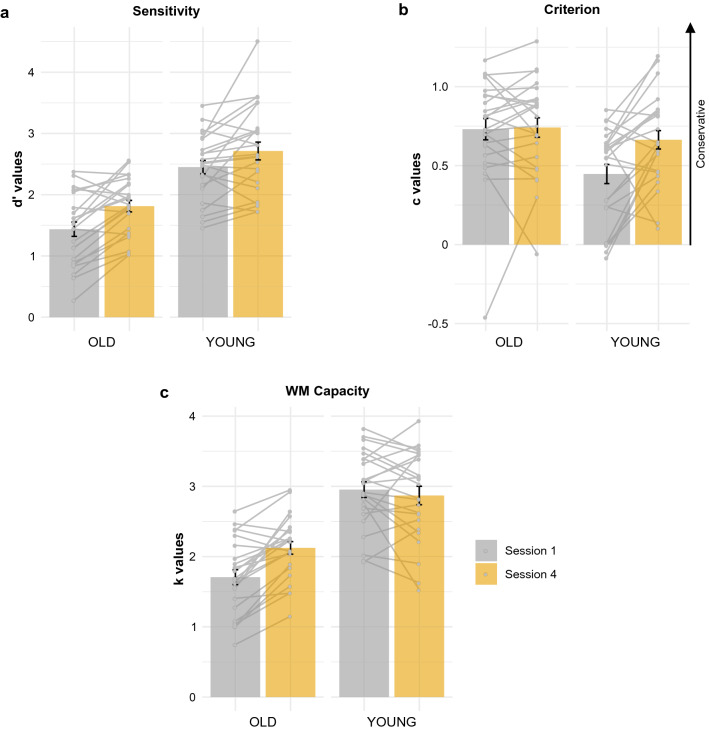
Behavioral results. (**a**) Mean sensitivity, (**b**) criterion and (**c**) WM capacity at Session 1 and 4 for the two groups, with Load4. Thin lines represent single-subject data while vertical bars represent standard errors.

Both groups thus showed an enhanced discrimination ability after practice, with an overall better sensitivity exhibited by young adults across sessions (Fig. 2a).

### Criterion (c)

#### Session 1 vs Session 4

From the main ANOVA, a main effect of Session (*F*(1, 46) = 8.383, *p* = 0.006, η_p_^2^ = 0.154) and a Session × Age interaction (*F*(1, 46) = 6.921, *p* = 0.012, η_p_^2^ = 0.131) were significant. First, to characterize the behavioral approach as conservative, neutral or liberal, one-sample t-tests against zero (i.e. a neutral approach) were performed for each group at each session. The t-tests resulted significant in both groups and both at Session 1 and 4, revealing that all participants exhibited a conservative behavior (i.e. c values significantly more positive than zero. Old: Session 1, *t*(23) = 10.849, *p*_adjusted_ < 0.001, *d* = 2.215; Session 4, *t*(23) = 12.015, *p*_adjusted_ < 0.001, *d* = 2.453. Young: Session 1, *t*(23) = 7.344, *p*_adjusted_ < 0.001, *d* = 1.499; Session 4, *t*(23) = 11.405, *p*_adjusted_ < 0.001, *d* = 2.328). The subsequent follow-up comparisons revealed that the Young group became more conservative across the practice period (*t*(23) = − 3.574, *p* = 0.002, *d* = 0.745). For the Old group, no difference was instead significant between Session 1 and 4 (*t*(23) = 0.243, *p* = 0.810, *d* = 0.043). Moreover, the two groups differed in their criterion at Session 1 (*t*(46) = 3.128, *p*_adjusted_ = 0.006, *d* = 0.922), where the Old group was more conservative, but not at Session 4 (*t*(46) = 0.917, *p*_adjusted_ = 0.364, *d* = 0.270) (Fig. 2b).

Young participants thus became more conservative as a result of practice, similar to the older age group.

### WM capacity (k)

#### Session 1 vs Session 4

The mixed ANOVA on k values at Load4 revealed significant main effects of Session (*F*(1, 46) = 8.172, *p* = 0.006, η_p_^2^ = 0.151) and Age (*F*(1, 46) = 47.097, *p* < 0.001, η_p_^2^ = 0.506), and a significant Session × Age interaction (*F*(1, 46) = 18.343, *p* < 0.001, η_p_^2^ = 0.285). From the Session × Age interaction, higher WM capacity of the Young with respect to the Old group was evident at both Session 1 (*t*(46) = − 8.107, *p*_adjusted_ < 0.001, *d* = 2.391) and 4 (*t*(46) = − 4.69, *p*_adjusted_ < 0.001, *d* = 1.383). Moreover, in young participants there was no significant improvement across sessions (*t*(23) = 0.904, *p* = 0.376, *d* = 0.188). In the Old group, an improvement across sessions was instead evident (*t*(23) =  − 5.799, *p* < 0.001, *d* = 1.209).

In summary, practice improved older participants’ WM capacity, although they never reached that of young participants (Fig. 2c).

### Session 2 vs Session 3

There was no difference in both groups for discrimination ability, criterion and WM capacity measures between Session 2 and Session 3.

### ERPs

The main mixed ANOVA showed significant main effects of Session (*F*(1, 46) = 4.663, *p* = 0.036, η_p_^2^ = 0.092), Load (*F*(2, 92) = 20.241, *p* < 0.001, η_p_^2^ = 0.306), Latency (*F*(2, 92) = 19.558, *p* < 0.001, η_p_^2^ = 0.298) and Age (*F*(1, 46) = 5.514, *p* = 0.023, η_p_^2^ = 0.107). Significant interactions emerged for Latency × Age (*F*(2, 92) = 4.694, *p* = 0.011, η_p_^2^ = 0.093), Session × Latency × Age (*F*(2, 92) = 5.142, *p*_adjusted_ = 0.009 as *G–G* ε = 0.872, η_p_^2^ = 0.101), Load × Latency (*F*(4, 184) = 32.034, *p*_adjusted_ < 0.001 as *G–G* ε = 0.744, η_p_^2^ = 0.411) and Session × Load × Latency × Age (*F*(4, 184) = 2.866, *p*_adjusted_ = 0.030 as *G–G* ε = 0.798, η_p_^2^ = 0.059).

To further investigate the four-way interaction, post-hoc comparisons were separately performed in each group, session and latency (i.e. N1, N2pc, CDA) through a series of pairwise comparisons run over consecutive 20-ms steps. Each pairwise comparison separately tested Load1 vs Load2 and Load2 vs Load4 conditions within-session. Only differences between conditions lasting for at least two consecutive 20-ms windows were considered reliable (see Methods for details).

**Figure 3:**
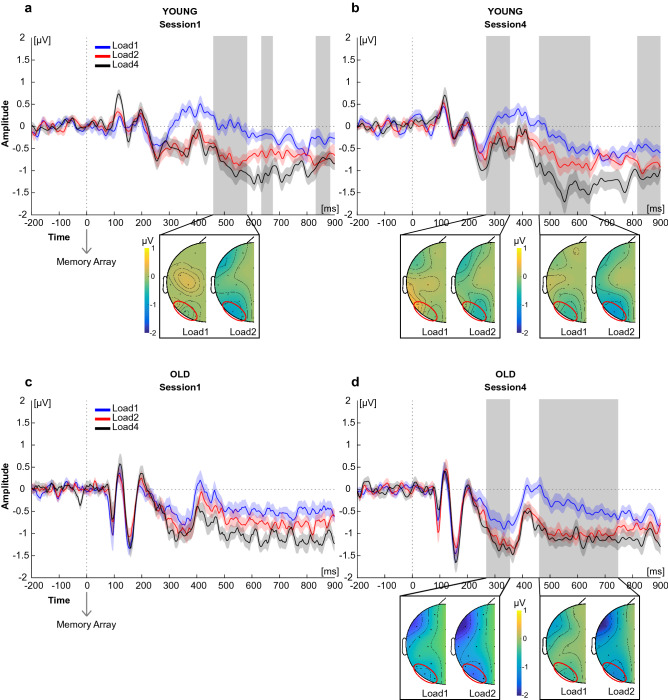
Grand average difference (contralateral minus ipsilateral) waveforms and topographical maps as a function of target load across sessions. (**a**) Young—Session 1. (**b**) Young—Session 4. (**c**) Old—Session 1. (**d**) Old—Session 4. Shaded areas represent standard errors at each time point. Grey squares indicate the time windows of significant difference between Load1 and Load2. Data of significant difference are projected over one hemisphere only, as target side was collapsed. Red circles indicate the ROI considered (P7/8, PO7/8, O1/2).

In Session 1 (Fig. 3a and Supplementary Fig. 1), young adults showed a significant difference between Load1 and Load2 only in the CDA latency (from 460 to 580 ms, from 640 to 680 ms and from 840 to 880 ms; all *t*s ≥ 2.490, all *p*s_adjusted_ ≤ 0.038, all *d*s ≥ 0.508), with Load2 exhibiting larger negative amplitude than Load1, while no difference emerged in the N1 and N2pc time windows (all *t*s ≤ 4.493, all *p*s_adjusted_ ≥ 0.056, all *d*s ≤ 0.503). There was instead no significant difference between Load2 and Load4 in any latency range (all *t*s ≤ 2.666, all *p*s_adjusted_ ≥ 0.179, all *d*s ≤ 0.544; see Supplementary Table 3 for full statistical details). In Session 4 (Fig. 3b and Supplementary Fig. 1), the mean amplitude for Load1 and Load2 significantly differed again in the CDA latency (from 460 to 640 ms and from 820 to 900 ms; all *t*s ≥ 2.291, all *p*s_adjusted_ ≤ 0.049, all *d*s ≥ 0.468), but also in the N2pc latency (from 270 to 350 ms; all *t*s ≥ 2.760, all *p*s_adjusted_ ≤ 0.016, all *d*s ≥ 0.516), always with Load2 having larger negative values than Load1, while they did not differ in the N1 (all *t*s ≤ 0.158, all *p*s_adjusted_ ≥ 0.989, all *d*s ≤ 0.032). Still no difference emerged from the comparison between Load2 and Load4 (all *t*s ≤ 3.392, all *p*s_adjusted_ ≥ 0.107, all *d*s ≤ 0.692; see Supplementary Table 4 for full statistical details).

For the older sample, in Session 1 (Fig. 3c and Supplementary Fig. 2) no significant differences were evident in any latency both for Load1 vs Load2 and Load2 vs Load4 (all *t*s ≤ 2.919, all *p*s_adjusted_ ≥ 0.057, all *d*s ≤ 0.596; see Supplementary Table 5 for full statistical details). In contrast, in Session 4 (Fig. 3d and Supplementary Fig. 2) there was a significant difference between Load1 and Load2 in the N2pc (from 270 to 350 ms; all *t*s ≥ 2.387, all *p*s_adjusted_ ≤ 0.041, all *d*s ≥ 0.487) and in the CDA latency (from 460 to 740 ms; all *t*s ≥ 2.692, all *p*s_adjusted_ ≤ 0.020, all *d*s ≥ 0.549), with larger negative amplitude for Load2 than Load1, while they did not differ in the N1 (all *t*s ≤ 1.856, all *p*s_adjusted_ ≥ 0.127, all *d*s ≤ 0.379). There was still no difference between Load2 and Load4 in any latency (all *t*s ≤ 2.272, all *p*s_adjusted_ ≥ 0.251, all *d*s ≤ 0.464; see Supplementary Table 6 for full statistical details).

In sum, whereas the CDA was already modulated by memory load during Session 1 in young adults (at least when one versus two targets when presented), practice anticipated such distinction during the N2pc range. Conversely, in the old group the two lowest memory loads (one versus two targets) differed only at Session 4, both in N2pc and CDA latency.

For the sake of completeness, further post-hoc comparisons were performed on the difference computed by subtracting Load1 from Load2 amplitude between sessions (Supplementary Table 7). A significant effect emerged only for a short interval of the CDA for older participants (from 460 to 500 ms).

### Control analysis: number of eye movements

Due to the lateralized nature of the task, we tested whether the improvements observed were related to an increase in eye movements towards the targets. Thus, the numbers of trials contaminated by horizontal eye activity (measured by the EEG) were compared between Session 1 and Session 4 via a t-test. No significant difference was observed between sessions in the number of eye movements in both groups (Young: *t*(23) = 0.243, *p* = 0.811, *d* = 0.050; Old: *t*(23) = − 0.810, *p* = 0.426, *d* = 0.165). Moreover, the difference in eye movements between Session 1 and Session 4 did not correlate (Pearson’s correlation) to any significant pre/post difference of any of the behavioral and EEG measures that were used (all *p*s > 0.156; see Supplementary Table 8 for full statistical details), thus further suggesting that the observed results were not due to an increase in the number of saccades.

## Discussion

This study investigated the effects of task repetition in aging by administering a DMTS task over four consecutive days. Our behavioral and EEG results provide new information on the mechanisms associated with learning in aging.

First, task repetition improved the sensitivity threshold of older participants, as indicated by an increase in the ability to distinguish change from no-change trials (d’). At the electrophysiological level we observed that the amplitude of the N2pc was modulated by memory load only in Session 4. Because of these results, and given previous findings indicating an association between sensitivity (as measured via d’) and the N2pc^81^, extensively proven to be a neural marker of attention individuation (e.g.^53,82^; but for alternative interpretations see the discussion in^83^), we suggest that in our experimental design d’ might reflect a behavioral component of performance (change sensitivity) that is tightly linked to target individuation. Therefore, our results would indicate that, following task repetition, older participants became more proficient in individuating the colored targets presented in the memory display together with irrelevant material (i.e. the grey dots), as reflected by the presence of a load-related modulation in the N2pc only during Session 4. This, in turn, allowed for a better sensitivity when a change in the stimulus display took place, as reflected by a practice-related increase of d’ values.

Previous electrophysiological studies^15,84,85^ have systematically linked the age-related attenuation of the N2pc to an impairment in the ability to individuate relevant items, which in turn negatively affects the performance of the elderly in tasks relying on such cognitive process (e.g. visual search, enumeration, DMTS). Hence, the present results appear promising as they reveal that the mechanism of attention individuation can be efficiently improved even through task repetition over few sessions.

Second, the results on older adults showed higher k values after task repetition (i.e. from ~ 1.7 in Session 1 to ~ 2.1 in Session 4). At the neural level, we found that the CDA response was modulated by memory load only after learning in the elderly. Similarly to the N2pc, the typical numerosity-related modulation of the CDA, considered as the neural counterpart of the lower vWM capacity exhibited by older participants in this task, is also reduced by aging^15,55^. In the present study, results indicated that while the CDA amplitude was not modulated by the number of targets before practice (when k values were set between one and two elements, i.e. ~ 1.7), it started to index one versus two memory load during the last session (when k values reached ~ 2.1 items, which would in turn explain why no difference is found between Load2 and 4). Given that the CDA has been linked to vWM capacity measured through k values in DMTS (see^86^ for a review), these results support the use of task repetition as a tool to improve vWM capacity in aging individuals when performing a DMTS.

Task repetition did not influence early perceptual abilities, as evidenced by no practice-related effects in the N1^49,50^. We hypothesize that this might be due to the task used here, because some previous studies^87,88^ using different tasks showed that changes in the N1 were evident in aging individuals following a visual perceptual training, so when perceptual abilities were specifically taxed (with a beneficial transfer effect to vWM).

Some effects of task repetition were evident also in young adults. Similar to older participants, at the behavioral level, task repetition enhanced the discrimination ability (d’), while EEG data revealed a load-dependent modulation of the N2pc only in the last session (similarly to what observed in old adults). This result seems to further underline the potential link between N2pc and d’ measures. In contrast, we did not find an improvement in vWM capacity, as measured through k values, which remained around three elements across all sessions. The absence of vWM improvement was in line with the electrophysiological data, where the CDA was modulated by target numerosity both before and after task repetition in young participants. Accordingly, the load-dependent modulations (for both the N2pc, in Session 4, and the CDA, in Sessions 1 and 4) were significantly different only between one and two targets. No difference was instead observed in the CDA amplitude between two and four memory loads, which could be associated with the vWM capacity estimate plateauing at three items in young participants (i.e., the typical vWM capacity limits measured in previous studies, see^39,89^; but see^90^). As for old adults, practice had no influence on early perception (no effect on the N1).

Further between-sessions comparisons on the N2pc and the CDA (see Supplementary table 7) revealed a trend of the results compatible with that observed from the main analyses, even though reaching statistical significance only for the CDA of old adults (and in a short time interval). The fact that no significant effects were found when comparing the N2pc between sessions is likely due to a lack of statistical power, as (1) overall, the N2pc is characterized by a smaller amplitude and a shorter duration compared to the CDA, (2) the absence of N2pc modulation is not unusual in DMTS tasks (e.g.^91^), probably because there is an overlap between the shorter-lasting N2pc and the more-sustained CDA onset, and (3) there may be task-unrelated inter-session variability in the EEG signal^92^. More in general, the present results are similar to previous research indicating a between-sessions effect in behavioral measures mainly reflected in a within-session load-related modulation of EEG data^93^.

Finally, repetition practice had an influence also on the decision bias (c) adopted by young participants. While both age groups showed an overall conservative attitude when performing the task (i.e. a tendency to answer ‘same’), only young participants strengthened such bias across sessions. It is not clear why task repetition should impact the response bias only in young adults. We speculate that this may be related to a decrease in alertness as a consequence of task repetition (as observed in vigilance tasks^94,95^).

Three limits of the present study should be noted. First, as there was no follow-up assessment, the duration of the effects found in the study is currently unknown. Second, the study was not designed to consider benefit generalization to other untrained tasks/domains (see^32,33,96,97^ for examples in this direction), being specifically focused on learning by task repetition. However, the outcomes of future EEG studies on task repetition may reveal useful hints when designing long-term WM training programs that specifically aim at an overall enhancement of WM abilities, and they could be considered as a biomarker for training success. For instance, such interventions could be designed to selectively train the cognitive processes that were mostly susceptible to changes, as reflected by the EEG measures from procedures similar to the present one. Third, we interpreted learning-related effects on the cognitive mechanisms subserving vWM based on the results obtained through EEG within-session comparisons (for the reasons outlined in the Statistical Analysis section), which might be considered an implicit way to draw conclusions in light of the fact that there are no strong between-sessions effects.

To conclude, the present study showed that task repetition is beneficial for various aspects related to vWM in healthy aging. Specifically, in older adults learning by task repetition boosted the mechanism of target individuation, presumably allowing individuals to retain more elements in the vWM buffer. According to the literature, age-related vWM reduction is due to changes in early perception^5–7,9,10^, attentive selection^9,11–16^ and/or short-term storage^17–19^. The current results thus reveal that improvements in the last two mechanisms (i.e. attention individuation and storage capacity) can be achieved already after two sessions of task repetition. This information may be instrumental for the design of future research on complex long-term interventions, which aim at generalizing training gains to different cognitive domains.

## Supplementary information

Supplementary Information

## Data Availability

Neither the data nor the materials have been made available on a permanent third-party archive; however, requests for the data or materials can be sent via email to the corresponding author.
