# Supplementary material for: Learning by task repetition enhances object individuation and memorization in the elderly
Source: Sci Rep. 2020 Nov 17;10:19957. doi: 10.1038/s41598-020-75297-x (PMC7673120; doi:10.1038/s41598-020-75297-x)
Supplement: Supplementary file 1 — Supplementary Information [file 41598_2020_75297_MOESM1_ESM.pdf]

Learning by task repetition enhances object individuation and memorization in the elderly

Chiara F. Tagliabue*[1], Sara Assecondi[2], Giulia Cristoforetti[1,3] and Veronica Mazza[1]

[1]Center for Mind/Brain Sciences (CIMeC) – University of Trento

[2]School of Psychology and Centre for Human Brain Health (CHBH), University of Birmingham

[3]Department of Experimental Psychology, University of Ghent

***Corresponding Author**

Chiara F. Tagliabue

chiara.tagliabue@unitn.it

Center for Mind/Brain Sciences (CIMeC) – University of Trento

Corso Bettini 31, 38068 Rovereto (TN), Italy

Numerical similarity analyses

Sensitivity (d')

The mixed ANOVA showed significant main effects of Session ($F(1, 46) = 20.744$, $p < .001$, $\eta_p^2 = .311$) and Age ($F(1, 46) = 36.349$, $p < .001$, $\eta_p^2 = .441$). Numerical similarity was not significant either as main effect or in interaction with other factors: Numerical similarity ($F(1, 46) = 3.200$, $p = .080$, $\eta_p^2 = .065$); Numerical similarity * Group ($F(1, 46) = 1.956$, $p = .169$, $\eta_p^2 = .041$); Session * Numerical similarity ($F(1, 46) = .554$, $p = .460$, $\eta_p^2 = .012$); Session * Numerical similarity * Group ($F(1, 46) = .141$, $p = .709$, $\eta_p^2 = .003$).

Criterion (c)

The mixed ANOVA showed a significant main effect of Session ($F(1, 46) = 9.495$, $p = .003$, $\eta_p^2 = .171$) and a significant Session * Group interaction ($F(1, 46) = 6.271$, $p = .016$, $\eta_p^2 = .120$). Numerical similarity was not significant either as main effect or in interaction with other factors: Numerical similarity ($F(1, 46) = .696$, $p = .409$, $\eta_p^2 = .015$); Numerical similarity * Group ($F(1, 46) = .142$, $p = .708$, $\eta_p^2 = .003$); Session * Numerical similarity ($F(1, 46) = 2.536$, $p = .118$, $\eta_p^2 = .052$); Session * Numerical similarity * Group ($F(1, 46) = .005$, $p = .945$, $\eta_p^2 = .000$).

WM Capacity (k)

The mixed ANOVA showed significant main effects of Session ($F(1, 46) = 7.846$, $p = .007$, $\eta_p^2 = .146$) and Group ($F(1, 46) = 47.077$, $p < .001$, $\eta_p^2 = .506$) and a significant Session * Group interaction($F(1, 46) = 17.630$, $p < .001$, $\eta_p^2 = .277$). Numerical similarity was not significant either as main effect or in interaction with other factors: Numerical similarity (F(1,

46) = 1.608, p = .211, η_p^2 = .034); Numerical similarity * Group (F(1, 46) = .676, p = .415, η_p^2 = .014); Session * Numerical similarity (F(1, 46) = .681, p = .413, η_p^2 = .015); Session * Numerical similarity * Group (F(1, 46) = .002, p = .965, η_p^2 = .000).

Supplementary Table 1.

Session 1		
**Variable**	**F**	**p**
d'	.397	.532
c	.431	.515
k	.081	.777
N1 Load1	4.308	.044
N1 Load2	5.620	.022
N1 Load4	4.667	.036
N2pc Load1	.009	.926
N2pc Load2	.195	.661
N2pc Load4	2.387	.129
CDA Load1	5.909	.019
CDA Load2	1.361	.249
CDA Load4	.032	.860
**Session 4**		
**Variable**	**F**	**p**
d'	3.729	.060
c	.077	.782
k	4.413	.041
N1 Load1	6.938	.01
N1 Load2	2.154	.149
N1 Load4	3.678	.061
N2pc Load1	2.303	.136
N2pc Load2	.256	.615
N2pc Load4	1.121	.295
CDA Load1	.484	.490
CDA Load2	.200	.657
CDA Load4	1.109	.298

Statistical details of Levene's test for homogeneity of variances between the two groups (old, young) of all the behavioral and electrophysiological variables. The red values indicate conditions where the assumption is not met.

Supplementary Table 2.

Session 1				
	Young		Old	
Variable	Shapiro-Wilk	p	Shapiro-Wilk	p
d’	.976	.801	.971	.681
c	.885	.011	.836	.011
k	.958	.394	.970	.679
N1 Load1	.915	.046	.920	.059
N1 Load2	.957	.379	.972	.708
N1 Load4	.989	.993	.941	.176
N2pc Load1	.966	.571	.946	.224
N2pc Load2	.926	.081	.908	.032
N2pc Load4	.953	.313	.888	.012
CDA Load1	.968	.625	.961	.461
CDA Load2	.975	.791	.941	.175
CDA Load4	.978	.858	.981	.913
**Session 4**				
	Young		Old	
Variable	Shapiro-Wilk	p	Shapiro-Wilk	p
d’	.952	.302	.959	.418
c	.975	.782	.968	.611
k	.954	.328	.975	.794
N1 Load1	.924	.071	.951	.290
N1 Load2	.934	.117	.965	.550
N1 Load4	.964	.520	.948	.245
N2pc Load1	.946	.219	.982	.929
N2pc Load2	.951	.284	.981	.918
N2pc Load4	.973	.744	.990	.996
CDA Load1	.979	.870	.969	.640
CDA Load2	.933	.113	.943	.189
CDA Load4	.964	.519	.962	.489

Statistical details of Shapiro-Wilk’s test for normal distribution of all the behavioral and electrophysiological variables. The red values indicate conditions where the assumption is not met.

Supplementary Table 3.

YOUNG – SESSION 1			
Load1 vs Load2			
msec	$p_{adjusted}$	t	d
N1			
120-140	0.687	-0.967	0.197
140-160	0.831	0.216	0.044
N2pc			
230-250	0.312	1.033	0.185
250-270	0.056	2.338	0.450
270-290	0.236	1.328	0.274
290-310	0.117	1.841	0.369
310-330	0.056	2.451	0.503
330-350	0.001	4.493	0.930
CDA			
460-480	0.019	3.080	0.629
480-500	0.012	3.435	0.701
500-520	0.019	3.145	0.642
520-540	0.003	4.114	0.840
540-560	0.003	4.089	0.835
560-580	0.002	4.784	0.977
580-600	0.111	1.828	0.373
600-620	0.022	2.898	0.592
620-640	0.092	1.955	0.399
640-660	0.037	2.534	0.517
660-680	0.038	2.490	0.508
680-700	0.233	1.316	0.269
700-720	0.061	2.230	0.455
720-740	0.037	2.625	0.536
740-760	0.127	1.723	0.352
760-780	0.455	0.796	0.162
780-800	0.992	0.010	0.002
800-820	0.249	1.243	0.254
820-840	0.092	1.985	0.405
840-860	0.020	2.996	0.612
860-880	0.037	2.580	0.527
880-900	0.138	1.649	0.337

YOUNG – SESSION 1			
Load2 vs Load4			
msec	$p_{adjusted}$	t	d
N1			
120-140	0.043691	-2.45972	0.502088
140-160	0.261777	1.150457	0.234836
N2pc			
230-250	0.954	-0.509	0.094
250-270	0.974	0.033	0.050
270-290	0.954	-1.235	0.209
290-310	0.954	-0.751	0.114
310-330	0.954	0.477	0.108
330-350	0.954	-0.263	0.025
CDA			
460-480	0.814	-0.286	0.058
480-500	0.771	0.390	0.080
500-520	0.468	1.114	0.227
520-540	0.506	1.013	0.207
540-560	0.566	0.884	0.180
560-580	0.671	0.608	0.124
580-600	0.179	1.936	0.395
600-620	0.179	2.666	0.544
620-640	0.179	2.038	0.416
640-660	0.179	2.017	0.412
660-680	0.179	1.974	0.403
680-700	0.323	1.395	0.285
700-720	0.179	2.347	0.479
720-740	0.179	2.284	0.466
740-760	0.236	1.677	0.342
760-780	0.323	1.407	0.287
780-800	0.181	1.871	0.382
800-820	0.179	1.980	0.404
820-840	0.764	0.446	0.091
840-860	0.639	-0.696	0.142
860-880	0.886	-0.145	0.030
880-900	0.596	0.798	0.163

Statistical details (FDR adjusted p values, ts, Cohen's ds) of each analyzed 20ms time window of Young adults during Session 1 (Load1 vs Load2 on the left, Load2 vs Load4 on the right). The red values indicate time points of significant difference (when ps < .05 and the significant difference persisted for at least two consecutive time windows, see Statistical analysis).

Supplementary Table 4.

YOUNG – SESSION 4					YOUNG – SESSION 4			
Load1 vs Load2					Load2 vs Load4			
msec	$p_{adjusted}$	t	d		msec	$p_{adjusted}$	t	d
N1					N1			
120-140	0.989	0.015	0.003		120-140	0.377	-1.355	0.277
140-160	0.989	-0.158	0.032		140-160	0.837	-0.209	0.043
N2pc					N2pc			
230-250	0.791	0.268	0.040		230-250	0.160	1.931	0.430
250-270	0.191	1.452	0.231		250-270	0.160	1.917	0.392
270-290	0.016	2.760	0.516		270-290	0.160	1.827	0.438
290-310	0.004	3.628	0.683		290-310	0.951	0.062	0.102
310-330	0.009	3.131	0.583		310-330	0.919	0.301	0.145
330-350	0.004	3.738	0.706		330-350	0.510	0.973	0.280
CDA					CDA			
460-480	0.011	3.389	0.692		460-480	0.193	1.564	0.319
480-500	0.015	2.999	0.612		480-500	0.107	2.081	0.425
500-520	0.049	2.291	0.468		500-520	0.107	2.188	0.447
520-540	0.004	4.268	0.871		520-540	0.107	2.410	0.492
540-560	0.007	3.782	0.772		540-560	0.055	3.392	0.692
560-580	0.015	2.938	0.600		560-580	0.107	2.240	0.457
580-600	0.015	2.969	0.606		580-600	0.169	1.673	0.342
600-620	0.047	2.348	0.479		600-620	0.107	2.713	0.554
620-640	0.015	2.927	0.597		620-640	0.107	2.508	0.512
640-660	0.015	2.983	0.609		640-660	0.131	1.851	0.378
660-680	0.109	1.786	0.365		660-680	0.107	2.092	0.427
680-700	0.466	0.778	0.159		680-700	0.131	1.885	0.385
700-720	0.544	0.616	0.126		700-720	0.110	2.020	0.412
720-740	0.217	1.329	0.271		720-740	0.226	1.401	0.286
740-760	0.124	1.678	0.343		740-760	0.201	1.503	0.307
760-780	0.109	1.773	0.362		760-780	0.107	2.625	0.536
780-800	0.067	2.113	0.431		780-800	0.107	2.150	0.439
800-820	0.090	1.934	0.395		800-820	0.373	0.908	0.185
820-840	0.008	3.637	0.742		820-840	0.373	0.943	0.192
840-860	0.004	4.192	0.856		840-860	0.373	0.981	0.200
860-880	0.015	3.102	0.633		860-880	0.373	1.046	0.213
880-900	0.047	2.381	0.486		880-900	0.373	0.977	0.199

Statistical details (FDR adjusted p values, ts, Cohen's ds) of each analyzed 20ms time window of Young adults during Session 4 (Load1 vs Load2 on the left, Load2 vs Load4 on the right). The red values indicate time points of significant difference (when ps < .05 and the significant difference persisted for at least two consecutive time windows, see Statistical analysis).

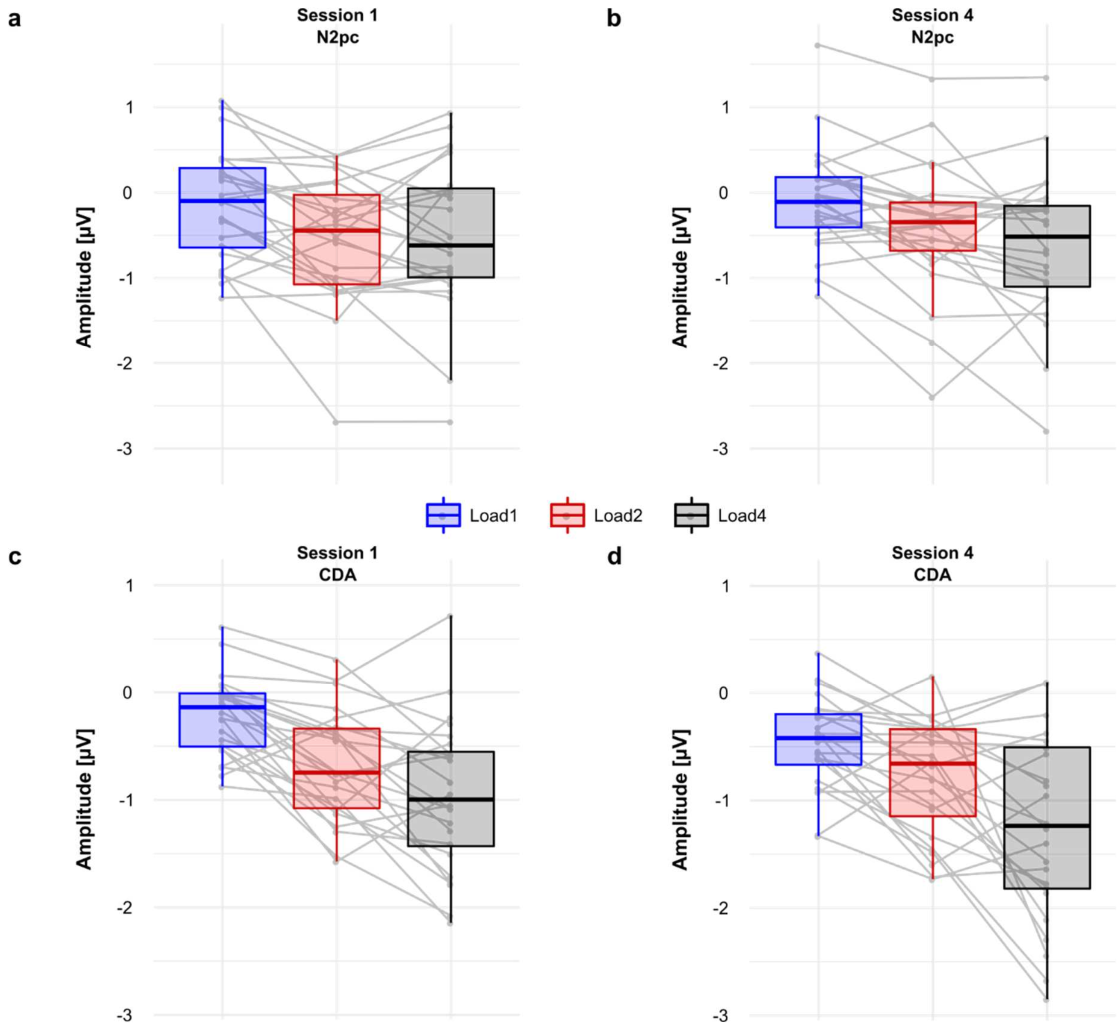

Supplementary Figure 1. Mean amplitudes of the N2pc (a, b) and the CDA (c, d) at Session 1 and Session 4 for Young Adults. Amplitudes were computed over the following time windows: 230-350 ms (N2pc) and 450-900 ms (CDA) (see Statistical analysis – *ERPs*). Thin lines represent single-subject data.

Supplementary Table 5.

OLD – SESSION 1					OLD – SESSION 1			
Load1 vs Load2					Load2 vs Load4			
msec	$p_{adjusted}$	t	d		msec	$p_{adjusted}$	t	d
N1					N1			
120-140	0.976	0.030	0.006		120-140	0.094	-2.097	0.428
140-160	0.145	-1.882	0.384		140-160	0.912	0.111	0.023
N2pc					N2pc			
230-250	0.627	0.650	0.133		230-250	0.861	-0.281	0.057
250-270	0.627	0.725	0.148		250-270	0.861	-0.177	0.036
270-290	0.724	-0.358	0.073		270-290	0.861	0.489	0.100
290-310	0.212	1.895	0.387		290-310	0.817	1.124	0.230
310-330	0.424	1.284	0.262		310-330	0.817	1.467	0.300
330-350	0.212	2.140	0.437		330-350	0.861	0.744	0.152
CDA					CDA			
460-480	0.079	2.286	0.467		460-480	0.096	2.134	0.436
480-500	0.278	1.141	0.233		480-500	0.038	3.257	0.665
500-520	0.079	2.279	0.465		500-520	0.096	2.188	0.447
520-540	0.187	1.478	0.302		520-540	0.140	1.775	0.362
540-560	0.216	1.338	0.273		540-560	0.240	1.364	0.279
560-580	0.057	2.919	0.596		560-580	0.187	1.546	0.316
580-600	0.125	1.797	0.367		580-600	0.055	2.929	0.598
600-620	0.079	2.365	0.483		600-620	0.247	1.266	0.258
620-640	0.119	1.897	0.387		620-640	0.067	2.542	0.519
640-660	0.121	1.852	0.378		640-660	0.062	2.654	0.542
660-680	0.053	3.123	0.637		660-680	0.348	0.990	0.202
680-700	0.079	2.526	0.516		680-700	0.247	1.249	0.255
700-720	0.152	1.632	0.333		700-720	0.121	1.889	0.386
720-740	0.106	1.995	0.407		720-740	0.247	1.283	0.262
740-760	0.216	1.331	0.272		740-760	0.558	0.594	0.121
760-780	0.082	2.166	0.442		760-780	0.062	2.744	0.560
780-800	0.079	2.345	0.479		780-800	0.079	2.371	0.484
800-820	0.081	2.214	0.452		800-820	0.156	1.682	0.343
820-840	0.079	2.520	0.514		820-840	0.119	1.983	0.405
840-860	0.152	1.626	0.332		840-860	0.032	3.614	0.738
860-880	0.025	3.712	0.758		860-880	0.121	1.912	0.390
880-900	0.327	1.001	0.204		880-900	0.079	2.334	0.476

Statistical details (FDR adjusted p values, ts, Cohen’s ds) of each analyzed 20ms time window of Old adults during Session 1 (Load1 vs Load2 on the left, Load2 vs Load4 on the right).

Supplementary Table 6.

OLD – SESSION 4			
Load1 vs Load2			
msec	$p_{adjusted}$	t	d
N1			
120-140	0.127	-1.856	0.379
140-160	0.127	-1.585	0.324
N2pc			
230-250	0.117	1.778	0.363
250-270	0.164	1.534	0.313
270-290	0.041	2.387	0.487
290-310	0.014	2.958	0.604
310-330	0.002	4.164	0.850
330-350	0.021	2.757	0.563
CDA			
460-480	0.000	5.028	1.026
480-500	0.000	5.381	1.098
500-520	0.002	3.856	0.787
520-540	0.001	4.510	0.921
540-560	0.001	4.264	0.870
560-580	0.000	4.845	0.989
580-600	0.000	4.839	0.988
600-620	0.006	3.410	0.696
620-640	0.001	4.141	0.845
640-660	0.006	3.260	0.665
660-680	0.020	2.692	0.549
680-700	0.009	3.070	0.627
700-720	0.006	3.277	0.669
720-740	0.006	3.271	0.668
740-760	0.172	1.494	0.305
760-780	0.076	1.990	0.406
780-800	0.048	2.274	0.464
800-820	0.496	0.692	0.141
820-840	0.358	1.004	0.205
840-860	0.068	2.070	0.423
860-880	0.147	1.613	0.329
880-900	0.383	0.923	0.188

OLD – SESSION 4			
Load2 vs Load4			
msec	$p_{adjusted}$	t	d
N1			
120-140	0.374	0.907	0.185
140-160	0.374	1.280	0.261
N2pc			
230-250	0.922	0.253	0.052
250-270	0.251	2.155	0.440
270-290	0.922	0.099	0.020
290-310	0.514	1.251	0.255
310-330	0.514	0.970	0.198
330-350	0.514	1.028	0.210
CDA			
460-480	0.626	0.639	0.130
480-500	0.802	0.254	0.052
500-520	0.626	0.715	0.146
520-540	0.525	1.427	0.291
540-560	0.626	0.621	0.127
560-580	0.675	0.468	0.095
580-600	0.569	-1.158	0.236
600-620	0.626	0.700	0.143
620-640	0.569	1.141	0.233
640-660	0.569	1.047	0.214
660-680	0.626	0.668	0.136
680-700	0.569	1.046	0.213
700-720	0.675	0.478	0.098
720-740	0.626	0.790	0.161
740-760	0.396	2.027	0.414
760-780	0.384	2.272	0.464
780-800	0.525	1.589	0.324
800-820	0.396	1.886	0.385
820-840	0.384	2.242	0.458
840-860	0.569	0.982	0.200
860-880	0.569	1.006	0.205
880-900	0.525	1.453	0.297

Statistical details (FDR adjusted p values, ts, Cohen's ds) of each analyzed 20ms time window of Old adults during Session 4 (Load1 vs Load2 on the left, Load2 vs Load4 on the right). The red values indicate time points of significant difference (when ps < .05 and the significant difference persisted for at least two consecutive time windows, see Statistical analysis).

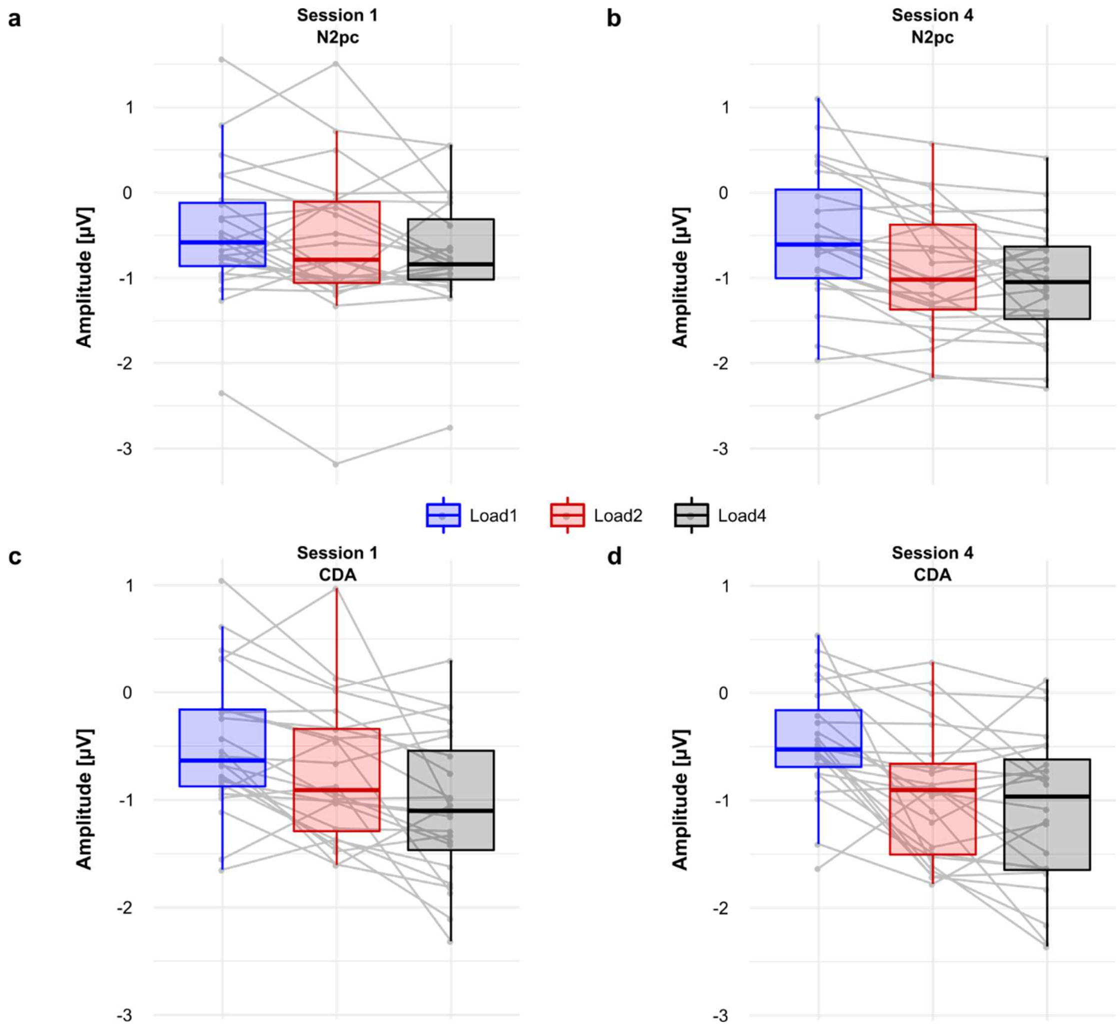

Supplementary Figure 2. Mean amplitudes of the N2pc (a, b) and the CDA (c, d) at Session 1 and Session 4 for Old Adults. Amplitudes were computed over the following time windows: 230-350 ms (N2pc) and 450-900 ms (CDA) (see Statistical analysis – *ERPs*). Thin lines represent single-subject data.

Supplementary Table 7.

YOUNG					OLD			
Session1 vs Session4 (Load2 – Load1 difference)					Session1 vs Session4 (Load2 – Load1 difference)			
msec	$p_{adjusted}$	t	d		msec	$p_{adjusted}$	t	d
N2pc					N2pc			
230-250	0.853	-0.643	0.173		230-250	0.705	0.594	0.121
250-270	0.853	-0.436	0.151		250-270	0.705	0.550	0.112
270-290	0.853	0.966	0.116		270-290	0.170	2.339	0.477
290-310	0.853	0.375	0.039		290-310	0.711	0.374	0.076
310-330	0.905	-0.120	0.069		310-330	0.705	0.994	0.203
330-350	0.853	-0.956	0.296		330-350	0.705	0.619	0.126
CDA					CDA			
460-480	0.961	0.516	0.105		460-480	0.031	3.169	0.647
480-500	0.961	-0.079	0.016		480-500	0.031	3.565	0.728
500-520	0.961	-0.338	0.069		500-520	0.318	1.635	0.334
520-540	0.961	-0.229	0.047		520-540	0.139	2.394	0.489
540-560	0.961	-0.598	0.122		540-560	0.168	2.193	0.448
560-580	0.961	-0.569	0.116		560-580	0.168	2.111	0.431
580-600	0.961	0.225	0.046		580-600	0.031	3.249	0.663
600-620	0.961	0.050	0.010		600-620	0.388	1.454	0.297
620-640	0.961	0.542	0.111		620-640	0.288	1.761	0.359
640-660	0.961	0.703	0.144		640-660	0.432	1.218	0.249
660-680	0.961	0.164	0.033		660-680	0.861	0.439	0.090
680-700	0.961	-0.231	0.047		680-700	0.388	1.394	0.285
700-720	0.961	-0.937	0.191		700-720	0.432	1.267	0.259
720-740	0.961	-0.424	0.087		720-740	0.759	0.713	0.146
740-760	0.961	-0.138	0.028		740-760	0.877	0.157	0.032
760-780	0.961	0.919	0.188		760-780	0.877	0.182	0.037
780-800	0.961	0.743	0.152		780-800	0.877	0.174	0.035
800-820	0.961	0.673	0.137		800-820	0.782	-0.633	0.129
820-840	0.961	0.139	0.028		820-840	0.615	-0.928	0.189
840-860	0.961	0.277	0.057		840-860	0.861	0.440	0.090
860-880	0.961	0.698	0.142		860-880	0.877	-0.225	0.046
880-900	0.961	0.574	0.117		880-900	0.877	0.174	0.036

Statistical details (FDR adjusted p values, ts, Cohen's ds) of each analyzed 20ms time window comparison between Session 1 and Session 4 on the Load2 – Load1 difference (Young on the left, Old on the right). The red values indicate time points of significant difference (when ps < .05 and the significant difference persisted for at least two consecutive time windows, see Statistical analysis).

Supplementary Table 8.

	Young		Old	
	**Pearson's r**	**p**	**Pearson's r**	**p**
Saccade Δ / d' Δ	-0.40	.853	.274	.195
Saccade Δ / c Δ	-.111	.606	.055	.797
Saccade Δ / c Δ	.022	.918	.299	.156
Saccade Δ / N2pc Δ	.142	.508	-.014	.949
Saccade Δ / CDA Δ	.041	.850	-.195	.361

Pearson's coefficients and p values for the correlations between the difference in number of saccades (Saccade Δ = Session 4 – Session 1) and the difference in behavioral (d' Δ / c Δ / k Δ = Session 4 – Session 1) end EEG (N2pc Δ / CDA Δ = Session 4 – Session 1) measures. N2pc Δ and CDA Δ between sessions were calculated on the difference between Load2 – Load1 at each session.
